# KG2ML: Integrating Knowledge Graphs and Positive Unlabeled Learning for Identifying Disease-Associated Genes

**DOI:** 10.1101/2025.03.17.25323906

**Published:** 2025-03-17

**Authors:** Praveen Kumar, Vincent T. Metzger, Swastika T. Purushotham, Priyansh Kedia, Cristian G. Bologa, Christophe G. Lambert, Jeremy J. Yang

**Affiliations:** 1 University of New Mexico (UNM), School of Medicine, Department of Internal Medicine, Translational Informatics Division, Albuquerque, New Mexico, USA

**Keywords:** KG2ML, ProteinGraphML, Positive and Unlabeled (PU) learning, PULSNAR, PULSCAR, gene-disease association

## Abstract

**Background:**

Biomedical knowledge graphs (KGs), such as the Data Distillery Knowledge Graph (DDKG), capture known relationships among entities (e.g., genes, diseases, proteins), providing valuable insights for research. However, these relationships are typically derived from prior studies, leaving potential unknown associations unexplored. Identifying such unknown associations, including previously unknown disease-associated genes, remains a critical challenge in bioinformatics and is crucial for advancing biomedical knowledge. Traditional methods, such as linkage analysis and genome-wide association studies (GWAS), can be time-consuming and resource-intensive. This highlights the need for efficient computational approaches to identify or predict new genes using known disease-gene associations. Recently, network-based methods and KGs, enhanced by advances in machine learning (ML) frameworks, have emerged as promising tools for inferring these unexplored associations. Given the technical limitations of the Neo4j Graph Data Science (GDS) machine learning pipeline, we developed a novel machine learning pipeline called KG2ML (Knowledge Graph to Machine Learning). This pipeline utilizes our Positive and Unlabeled (PU) learning algorithm, PULSNAR (Positive Unlabeled Learning Selected Not At Random), and incorporates path-based feature extraction from ProteinGraphML.

**Results:**

KG2ML was applied to 12 diseases, including Bipolar Disorder, Coronary Artery Disease, and Parkinson’s Disease, to infer disease-associated genes not explicitly recorded in DDKG. For several of these diseases, 14 out of the 15 top-ranked genes lacked prior explicit associations in the DDKG but were supported by literature and TINX (Target Importance and Novelty Explorer) evidence. Incorporating PULSNAR-imputed genes as positives enhanced XGBoost classification, demonstrating the potential of PU learning in identifying hidden gene-disease relationships.

**Conclusion:**

The observed improvement in classification performance after the inclusion of PULSNAR-imputed genes as positive examples, along with the subject matter experts’ (SME) evaluations of the top 15 imputed genes for 12 diseases, suggests that PU learning can effectively uncover disease-gene associations missing from existing knowledge graphs (KGs). By integrating KG data with ML-based inference, our KG2ML pipeline provides a scalable and interpretable framework to advance biomedical research while addressing the inherent limitations of current KGs.

## Background

Genes are the fundamental units of inheritance and play a critical role in determining an individual’s susceptibility to various conditions and disorders [1]. Mutations or genetic variations can disrupt normal biological processes, potentially leading to diseases. Identifying genes that are causally linked to specific genetic diseases is crucial for improving human health [2]. Such knowledge provides insights into the molecular mechanisms underlying diseases, which are essential for the development of effective diagnostic and therapeutic strategies. Moreover, understanding gene-disease associations enables the identification of at-risk individuals, allowing for early interventions to reduce the likelihood of disease onset and progression [3–5].

Identifying genes associated with specific diseases remains an open problem in bioinformatics [6,7]. Traditional approaches for disease-associated genes identification, such as linkage analysis and genome-wide association studies (GWAS), are often time-consuming and resource-intensive [8]. Consequently, the development of efficient computational methods to identify or predict novel genes using known disease-gene associations has become crucial. Network-based computational methods are widely employed to infer disease-gene associations [9]. These networks are constructed using biological and molecular prior knowledge, capturing complex relationships between entities, such as genes, proteins, and diseases [10]. Knowledge graphs (KGs) further enhance this approach by integrating diverse biological networks and ontologies into a unified, comprehensive framework. KGs leverage complex semantic relationships among entities to generate valuable insights [9,10]. Recent advancements in machine learning (ML) frameworks have expanded the application of ML algorithms to KGs for the identification and prediction of novel disease-associated genes [7–13]. Applying ML methods to KGs typically involves transforming the network of entities into a feature matrix through techniques such as knowledge graph embeddings [11,14] or path-based feature extraction methods [15,16]. These approaches allow the integration of heterogeneous data types and enhance the discovery of hidden patterns and associations.

In this study, we propose a machine learning pipeline called KG2ML (Knowledge Graph to Machine Learning) employing a Positive and Unlabeled (PU) learning algorithm and a path-based feature extraction method to derive features from the knowledge graph. The primary objective of this study is to identify genes potentially associated with diseases, even in the absence of explicit links between them in the knowledge graph. Since all entities and their relationships in a KG are derived from prior knowledge, the absence of a direct link between a gene and a disease does not necessarily indicate that the gene is unrelated to the disease. This motivated the use of PU learning instead of traditional binary classification based on positive and negative examples. Specifically, we utilized PULSNAR (Positive Unlabeled Learning Selected Not At Random) [17] as our PU learning algorithm, while the method for generating feature vectors for each gene was adapted from our previous work, ProteinGraphML [16].

### Data Distillery Knowledge Graph (DDKG)

The Data Distillery Knowledge Graph (DDKG) [18] has been developed as part of the Common Fund Data Ecosystem (CFDE) Data Distillery Partnership Project. This collaborative effort is led by the CFDE HuBMAP, SenNet, and Kids First teams from the University of Pittsburgh and the Children’s Hospital of Philadelphia (CHOP). Built on the Neo4j platform, the DDKG aims to integrate and distill data from multiple Common Fund data coordinating centers (DCCs), ensuring semantic interoperability to support a wide range of integrative biomedical research questions and scientific use cases, such as identifying novel relationships between biomedical entities. The DDKG schema is based on the Unified Medical Language System (UMLS) and utilizes the Unified Biomedical Knowledge Graph (UBKG) to provide a robust framework for the DDKG, with rigorous semantics and interoperability supported by the UMLS comprehensive metathesaurus, incorporating over 180 ontologies and standards.

## ProteinGraphML

ProteinGraphML[16] is a Python-based package designed to predict associations between diseases and protein-coding genes using a biomedical knowledge graph. The package employs XGBoost[19], a gradient-boosting machine learning algorithm, to estimate the likelihood of these associations. It utilizes the metapath approach to transform the complex, heterogeneous knowledge graph into a feature matrix, where rows represent proteins and columns correspond to feature vectors (either categorical or continuous variables). In the context of ProteinGraphML, a metapath is defined as a sequence of nodes and edges that form a path connecting a target protein to a disease. These metapaths capture the semantic information embedded in various path types between nodes, effectively translating the graph structure into features for machine learning models. This metapath approach allows ProteinGraphML to utilize the complex relationships within the biomedical knowledge graph, enhancing its ability to predict disease-protein associations [16].

### Positive Unlabeled Learning Selected Not At Random (PULSNAR)

Traditional binary classification techniques, which distinguish between positive and negative instances, are well-suited for fully labeled datasets. However, in a heterogeneous knowledge graph, the existence of a link between a disease and a gene indicates a known association, but the absence of a link does not necessarily imply a negative relationship. The gene with missing link might be positive whose association has not yet been established in studies. This leads to the issue of the lack of reliable negative examples for classification problems. This lack of reliable negative examples poses a significant challenge for traditional binary classification methods, potentially leading to biased results[20,21]. To address this limitation in KG2ML, we employed PULSNAR, a Positive and Unlabeled learning technique we developed specifically for PU datasets, where reliable negative examples are missing.

Our PULSNAR package offers flexible implementation, supporting both SCAR (Selected Completely At Random) and non-SCAR scenarios, depending on the nature of the data. The package includes two main algorithms: PULSCAR (Positive Unlabeled Learning Selected Completely At Random) and PULSNAR. PULSCAR assumes that labeled positive examples are randomly selected, independent of their attributes [22]. In contrast, PULSNAR operates under the SNAR (Selected Not At Random) assumption, where the selection of labeled positives is dependent on their attributes [17].

PULSNAR helps identify genes that lack explicit links to a disease in the KG but may still be associated with it. For a given disease, PULSNAR estimates the proportion (ɑ) of positive genes among those without explicit links to the disease in the KG and assigns a likelihood score to each unassociated gene, indicating its probability of being a positive gene for the disease. Thus, by integrating PULSNAR, KG2ML enhances the discovery of potential disease-gene associations, particularly in cases where explicit negative examples are unavailable or unreliable.

## Materials and Methods

To apply the KG2ML pipeline to gene and disease data, we constructed a subset of the DDKG, termed *CondensedKG*, which includes all relevant node types and corresponding relationship types required for this study. A feature matrix was generated by applying a path-based approach to the nodes and edges within the CondensedKG. The subsequent subsections detail the following: (1) the methodology for creating the CondensedKG, (2) the KG2ML workflow for generating the feature matrix, (3) the limitations and challenges associated with the Neo4j Graph Data Science (GDS) library, (4) the identification of positive genes among those without direct links to diseases in the CondensedKG, (5) the diseases selected to evaluate the KG2ML pipeline, and (6) the validation process for imputed genes. All code necessary for constructing the CondensedKG and KG2ML pipelines are available in our GitHub repository: https://github.com/unmtransinfo/cfde-distillery.

### DDKG to CondensedKG

**Figure 1:**
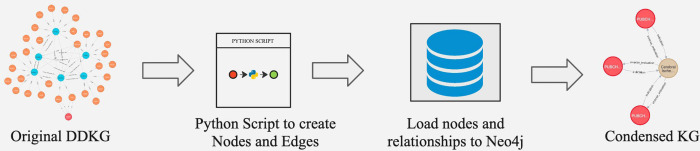
Overview of ETL workflow to generate CondensedKG from DDKG.

The DDKG integrates diverse biomedical data from multiple ontologies and DCCs, resulting in a highly complex and large-scale knowledge graph. Executing Cypher queries on such a large-scale knowledge graph to extract data for specific use cases can be resource-intensive and time-consuming. Additionally, the interpretability of the extracted data can be challenging due to its complexity. Given that our primary objective in this study is to identify gene-disease associations, we generated a subset of the DDKG, termed *CondensedKG,* to improve both data interpretability and query performance. CondensedKG comprises nodes representing genes, proteins, compounds, and diseases, along with edges that denote the relationships among these entities. Specifically, it includes 8 distinct node labels and 1,042 unique relationship types. Figure 1 illustrates the step-by-step process of generating CondensedKG from DDKG. This CondensedKG offers two key advantages: (1) it enhances data interpretability by providing a more focused and concise representation of gene-disease relationships, and (2) it significantly improves Cypher query execution times by reducing the number of nodes and edges compared to the full DDKG.

### KG2ML workflow

The comprehensive workflow of the KG2ML pipeline is illustrated in Figure 3. Before applying the PULSNAR method to gene-disease data for discovering genes lacking direct links to diseases within the CondensedKG, we first identify genes with direct links to diseases (positive instances) and genes without direct links (unlabeled instances). Subsequently, we generate feature vectors for both the positive and unlabeled gene sets.

#### 1) Label generation

The following Cypher query was used to select all genes for inclusion in the KG2ML pipeline for Parkinson’s disease. The same query was applied to all other diseases in our study by substituting their corresponding Concept Unique Identifiers (CUIs)(*d.CUI* value in this query). In this query, the first MATCH statement selects all gene nodes (positive) that are directly connected to the target disease node through a compound node. The second MATCH statement selects all other gene nodes (unlabeled) that are connected to positive genes (g1:Gene) via Experimental Factor Ontology (EFO) nodes (e:EFO). For the PULSNAR method, genes identified by the first MATCH statement (g1:Gene) were labeled as class 1, while those identified by the second MATCH statement (g2:Gene) were labeled as class 0.

MATCH (d:Disease)-[]-(c:Compound)-[]-(g1:Gene) WHERE d.CUI=‘C0030567’

MATCH (g1:Gene)-[]-(e:EFO)-[]-(g2:Gene)

RETURN DISTINCT g1.CUI as positive_genes_cui, g1.node_label as positive_genes_label, e.CUI as features, c.CUI as features_to_remove, g2.CUI as unknown_genes_cui, g2.node_label as unknown_genes_label

#### 2) Feature generation

All EFO nodes (e:EFO) connecting positive genes (g1:Gene) to unlabeled genes (g2:Gene) were used as features. For each gene, if it was connected to a specific EFO node, the corresponding feature value was set to 1; otherwise, it was set to 0. This encoding resulted in a feature matrix of size n × m for running the PULSNAR models, where n represents the number of unique positive and unlabeled genes, and m denotes the number of unique features (EFO nodes). Since most genes had relatively few nonzero values (1s) in their feature vectors, the feature matrix was converted into a Compressed Sparse Row (CSR) format, significantly reducing memory usage and enhancing computational efficiency for matrix operations and storage.

### HashGNN - Neo4j Graph Data Science (GDS)

The Neo4j Graph Data Science (GDS)[23] library offers machine learning pipelines for feature extraction from nodes and relationships, as well as for training supervised models to predict node properties and infer missing relationships. The library’s integration with Neo4j’s native graph storage and processing capabilities, combined with HashGNN’s theoretical ability to handle large-scale graphs efficiently, made it an attractive candidate for our biomedical knowledge graph analysis. Given the heterogeneous nature of our knowledge graph, CondensedKG, and the non-deterministic results produced by the Node2Vec node embedding algorithm—even when the randomSeed configuration parameter is specified—we exclusively utilized the HashGNN node embedding algorithm for analyzing DDKG in this study. HashGNN is particularly well-suited for embedding heterogeneous graphs and provides more deterministic results compared to algorithms like Node2Vec, especially when the randomSeed parameter is specified.

However, our experimental implementation revealed several limitations that made the HashGNN algorithm unsuitable for our KG2ML framework’s specific requirements explained in the KG2ML workflow section. While HashGNN offered efficient graph neural network operations through locality-sensitive hashing, its implementation in Neo4j GDS showed significant constraints when dealing with heterogeneous biomedical knowledge graphs. The algorithm’s performance degraded considerably when processing complex relationship types and multi-hop connections, which are characteristic of gene-disease associations. Additionally, HashGNN’s feature hashing mechanism, while efficient for homogeneous graphs, failed to adequately capture the semantic richness of different relationship types in our CondensedKG, leading to the loss of critical information about gene-disease interaction patterns.

Furthermore, beyond the HashGNN-specific limitations, Neo4j GDS presented additional challenges that affected our framework’s effectiveness. Its inability to efficiently perform batch operations for querying and processing large-scale heterogeneous knowledge graphs significantly hindered performance. Moreover, the library’s node embedding features, including HashGNN and other algorithms like FastRP and Node2Vec, lacked the flexibility required for our specific use case of disease-gene association prediction. In particular, the meta path-based approach we required for generating meaningful feature vectors that capture the complex biological relationships between genes, proteins, and diseases was not directly supported by Neo4j GDS’s embedding capabilities. These technical constraints, especially HashGNN’s limitations in handling heterogeneous graphs, led us to develop a custom path-based feature generation method that better met our specific needs for disease-gene association prediction. Therefore, this manuscript does not compare the performance of the KG2ML pipeline with the Neo4j GDS machine learning pipeline.

### Diseases used for testing the KG2ML module

Table 1 lists the diseases/conditions which were selected to evaluate the KG2ML pipeline. These well-defined diseases and conditions were specifically selected based on the following considerations: (1) availability of sufficient data, (2) biomedical importance and interest to researchers, and (3) because they are responsible for significant disease burden. Focusing on diseases with a large number of connections ensures a sufficient number of both positive and unlabeled examples, facilitating a robust evaluation of the KG2ML’s performance. Furthermore, the purposeful inclusion of a diverse range of medical conditions enabled a comprehensive evaluation of the module’s effectiveness across various diseases.

### Alpha estimation, identification of probable positive genes and classification performance

For all 12 diseases used in our experiment, we applied the SCAR-based algorithm, PULSCAR, because all positive genes for these diseases were of a single type—they were all connected to the target disease via compound nodes. Applying the non-SCAR-based method, PULSNAR, to SCAR data can lead to an overestimation of the fraction of positives among the unlabeled examples. The PULSCAR method estimated the proportion (α) of genes potentially associated with a given disease among those that lacked explicit links (unlabeled) in the CondensedKG. Using the estimated α value, PULSCAR generated calibrated probabilities for each unlabeled gene, enabling the identification of genes that were more likely to be associated with the target disease despite missing links in the CondensedKG.

In the absence of a definitive ground truth for negative genes, we employed two different modeling approaches to evaluate the performance of the XGBoost model. In the first approach, XGBoost was trained and tested using 5-fold cross-validation (CV), where all labeled positive genes were treated as class 1 and all unlabeled genes as class 0. In the second approach, XGBoost was trained and tested using 5-fold CV, but in this case, both labeled positive genes and probable positive genes identified using the PULSCAR method were treated as class 1, while the remaining unlabeled genes were treated as class 0. In both approaches, models were trained and tested using 5-fold CV for 40 iterations to estimate the 95% confidence interval (CI). This comparative analysis allowed us to assess the effectiveness of PULSCAR in distinguishing probable positive genes from truly unassociated genes.

Additionally, since all 12 datasets contained only labeled positive examples (with no explicitly labeled negative examples), we calculated recall for both models using only the labeled positive examples. Specifically, this evaluation aimed to determine whether incorporating probable positives identified by PULSCAR improved the model’s ability to recall known positive genes.

We also utilized the DEDPUL[24], TiCE[25], and KM methods[26] to estimate the proportion of positive genes among unlabeled genes. The publicly available implementations of the KM and TiCE methods are not optimized for large datasets, so running 40 iterations for all 12 diseases/conditions would have taken approximately 35–40 days. Therefore, these methods were only executed for 20 iterations.

### Validation of imputed genes

To validate the genes imputed by the PULSCAR method, we selected the top 15 genes with the highest calibrated probabilities for each of the 12 diseases. The calibrated probabilities were computed as the mean values obtained from 40 independent iterations of the PULSCAR model, ensuring robustness and reliability in the probability estimates. Validation was performed by a subject matter expert (SME) through a comprehensive review of published literature and the TINX database[27], a well-established resource for gene-disease associations. For each gene, the SME assessed its association with the corresponding disease based on available evidence. If a documented association was found in either the literature or the TINX database, the gene was classified as ‘Yes’ (associated). If no prior evidence of association was identified, it was classified as ‘No’ (not associated). This expert-driven validation process provided an external assessment of the PULSCAR model’s ability to identify disease-gene associations, further supporting its potential for uncovering previously unrecognized links in biomedical knowledge graphs.

## Results

Table 2 presents the α values estimated by the PULSCAR method for each of the conditions/diseases used to evaluate the KG2ML module. These α values represent the estimated proportion of genes that, despite lacking explicit links with a given disease in the knowledge graph, may potentially be associated with the condition. This suggests that a substantial fraction of unlabeled genes could have undiscovered associations with the diseases under study.

In Table 2, the column labeled ‘XGBoost only’ corresponds to Model 1, which was trained and tested using 5-fold CV. In this model, all labeled positive genes were treated as class 1, and all unlabeled genes were treated as class 0. The column labeled ‘XGBoost + PULSCAR’ corresponds to Model 2, which was also trained and tested using 5-fold CV. However, in Model 2, both labeled positive genes and probable positive genes identified by the PULSCAR method were treated as class 1, while the remaining unlabeled genes were treated as class 0. The recall of Model 2 showed significant improvement over Model 1, demonstrating that incorporating probable positives identified by PULSCAR enhances the model’s ability to predict known positive genes.

Table 3 presents a comparison of alpha estimates obtained using SCAR-based methods: PULSCAR, DEDPUL, KM1, KM2, and TiCE. Since KM methods select all positive and unlabeled instances to estimate the proportion, they yielded the same alpha values across iterations. Notably, in the absence of a known true alpha for each disease, the estimated values cannot be directly validated. However, the alpha estimates from different methods suggest that some genes without direct links to the disease in existing knowledge graphs may still be associated with the disease, warranting further investigation to confirm these potential associations.

Figure 3 presents the classification performance of two XGBoost-based models, highlighting the impact of incorporating PULSCAR-identified probable positive and negative genes into the classification models. Model 1 (labeled as “XGBoost only” and represented by the blue bars) was trained and tested using 5-fold cross-validation (CV). In this model, labeled positive genes were assigned to class 1, while all unlabeled genes were treated as class 0. This serves as a baseline model, operating under the assumption that all unlabeled genes are negative. Model 2 (labeled as “XGBoost + PULSCAR” and represented by the red bars) followed the same 5-fold CV procedure. However, in this model, both labeled positive genes and probable positive genes identified by PULSCAR were assigned to class 1, while the remaining unlabeled genes were considered class 0. By incorporating the probable positive and negative genes inferred by PULSCAR, we observed a substantial improvement across all classification performance metrics across all datasets. This demonstrates the effectiveness of PULSCAR in distinguishing between positive and negative genes within the unlabeled set, thereby enhancing overall predictive accuracy.

Table 4 presents the top 15 genes with the highest calibrated probabilities of association for each of the 12 diseases analyzed in this study. Validation through a comprehensive review of existing scientific literature and the TINX database confirmed that many of these top-ranked genes are indeed associated with their respective diseases. This result highlights the effectiveness of PU learning in identifying potential disease-gene associations that are not explicitly represented in the knowledge graph.

## Discussion

Biomedical knowledge graphs integrate information from existing literature and databases, representing entities such as genes, diseases, and compounds as nodes and their associations as edges. However, a common limitation of these knowledge graphs is data incompleteness, as they often lack certain entities or relationships due to gaps in current knowledge [28]. In this study, we utilized *CondensedKG*, a subset of the DDKG, which aggregates nodes and edges contributed by various DCCs. Despite its comprehensive nature, *CondensedKG*—like other knowledge graphs—remains subject to data gaps and missing associations. However, as demonstrated in our study, machine learning methods, particularly PU learning, can effectively uncover such missing associations. The α values estimated by the KG2ML pipeline for all 12 diseases in our study highlight the efficacy of PU learning in detecting genes that are not explicitly linked to diseases in the knowledge graph. However, not all imputed genes could be validated due to the novelty of these associations, as no prior studies have yet established these gene-disease relationships. The lack of validation for certain gene-disease pairs does not necessarily indicate that the associations identified by the PU learning method are incorrect; rather, it suggests the potential for novel discoveries that warrant further investigation.

Knowledge graph embedding algorithms map entities and relationships from a knowledge graph into a continuous vector space while preserving the graph’s semantic and structural properties [29]. These embeddings enable efficient computation and facilitate machine learning tasks such as link prediction and entity classification. However, as outlined in the subsection “HashGNN - Neo4j Graph Data Science (GDS),” Neo4j’s graph embedding algorithms can exhibit non-deterministic behavior despite setting a random seed, which poses challenges for reproducibility. To address this limitation, we adopted a path-based embedding technique from our previous work, ProteinGraphML, ensuring more consistent and interpretable feature vector representations. This approach not only enhanced reproducibility but also provided embeddings better suited for the downstream PU learning method, PULSCAR.

Across all datasets, Model 2 consistently outperformed Model 1 across all evaluation metrics (Figure 3 and Table 2). This improvement suggests that the application of PU learning effectively differentiated probable positive and negative examples within the unlabeled set, thereby refining the dataset. The improved data quality enabled the model to learn more precise decision boundaries, resulting in superior classification performance compared to the baseline model (Model 1), which treated all unlabeled examples as negative instances (class 0). This finding is consistent with prior studies demonstrating that reducing noise in the labels improves model performance [30]. A review of PU learning in bioinformatics and computational biology [31] also found that studies reported performance improvements when using PU learning methods. The significant improvement in recall, as shown in Table 2, further supports the accuracy of our PU method, PULSCAR, in identifying probable positives within the unlabeled set. Since the recall was calculated using only labeled positive examples, the results suggest that the probable positives identified by PULSCAR were predominantly true positives rather than false positives. Our findings highlight the potential of PU learning to improve gene-disease association predictions, making it a valuable computational approach for advancing biomedical research.

### Limitations

To validate the genes identified by the KG2ML module, we referenced published scientific literature along with the TINX database. If a gene-disease association was neither documented in prior studies nor available in TINX, we could not confirm its validity based on the existing knowledge. Consequently, our findings should be interpreted as methodological advancements in identifying previously unrecognized disease-associated genes, rather than as definitive associations.

### Future work

In this study, we were unable to utilize the Neo4j Graph Data Science module due to its technical constraints, as outlined in the subsection “HashGNN - Neo4j Graph Data Science (GDS).” In future research, we aim to integrate a deterministic node embedding algorithm of Neo4j with our KG2ML pipeline. This investigation will evaluate the effectiveness of GDS-derived embeddings in enhancing PU learning performance for identifying disease-gene associations.

## Conclusions

Like other biomedical knowledge graphs, *CondensedKG*, despite being a subset of the comprehensive DDKG, lacks certain associations among existing entities such as genes, diseases, compounds, and proteins. Since all gene-disease associations in *CondensedKG* are derived from prior studies and established databases, genes explicitly linked to a disease can be considered positive genes for that disease. However, genes lacking such associations in the knowledge graph cannot be assumed to be negative genes, as their true relationships may simply be undiscovered. This characteristic makes PU learning particularly suitable for analyzing such data, as it is specifically designed to identify unknown associations in the absence of confirmed negative examples. The α values estimated by PULSCAR suggest that numerous missing gene-disease associations exist in *CondensedKG*, highlighting the potential for novel discoveries. However, these predicted associations require experimental validation to confirm their biological relevance. Manual validation of imputed genes by domain experts further demonstrates the potential of PU learning as a computational framework for advancing biomedical research. By integrating knowledge graph analysis with PU learning, the KG2ML pipeline presents a methodological advancement, providing a robust and scalable framework for uncovering novel gene-disease associations.

## Supplementary Material

Supplement 1

## Figures and Tables

**Figure 2:**
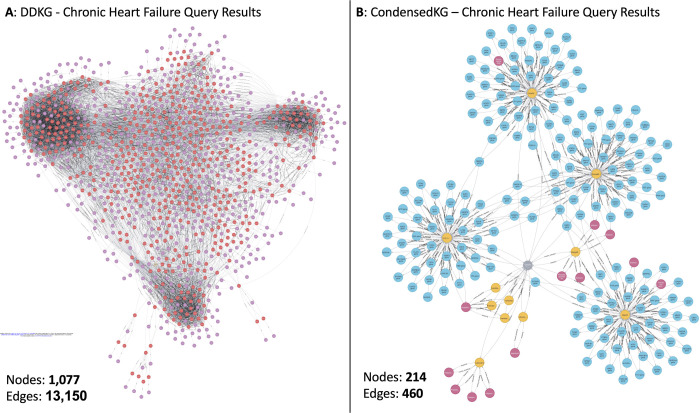
Comparison of Chronic heart failure cypher query between DDKG and CondensedKG

**Figure 3:**
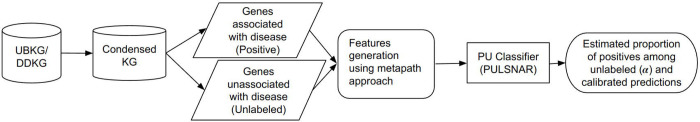
Comprehensive workflow of the KG2ML pipeline..

**Figure 3:**
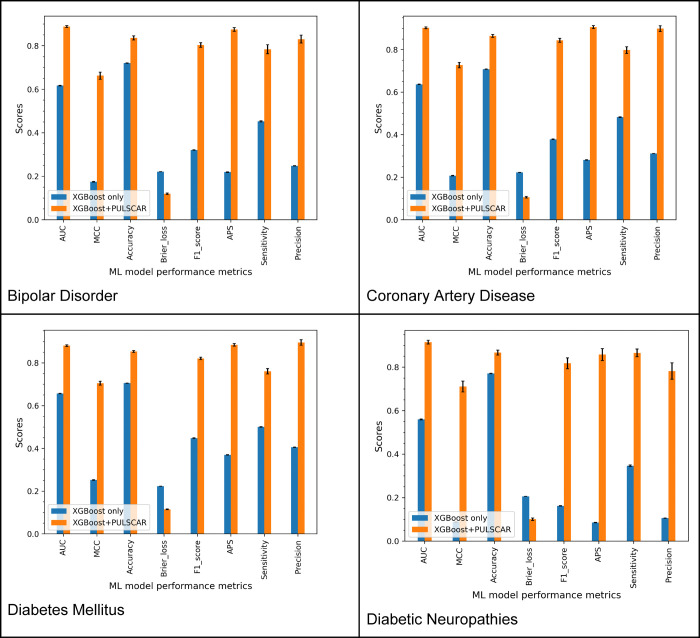

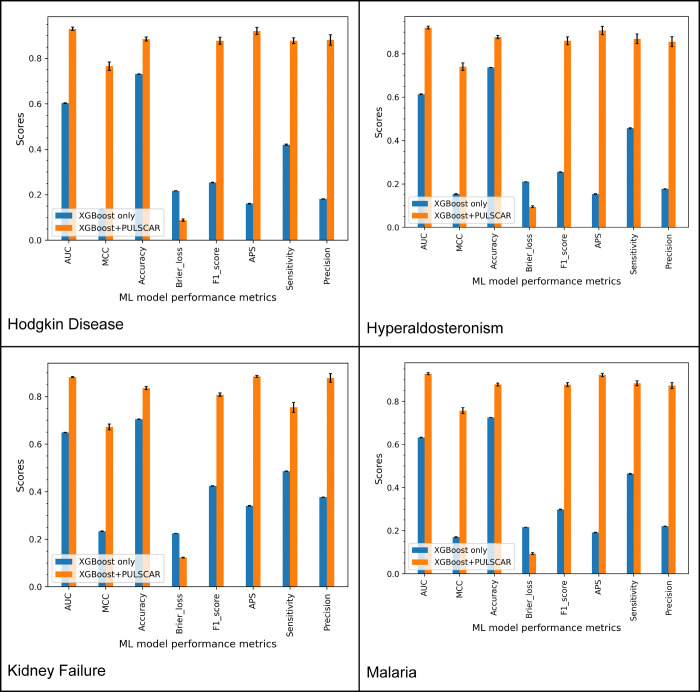

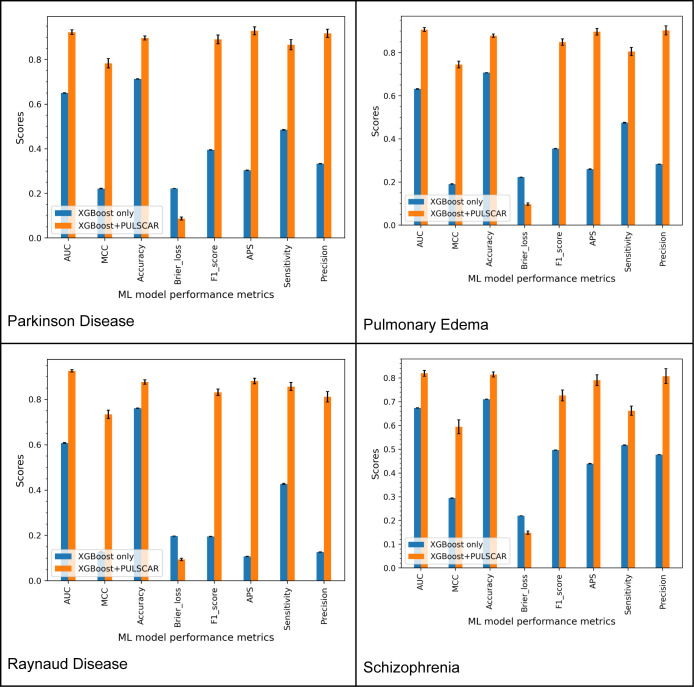
Classification performance of XGBoost models trained and tested on labeled positives as class 1 and unlabeled instances as class 0 (*‘XGBoost only’*) vs. labeled and PULSCAR-imputed probable positives as class 1 and probable negatives as class 0 (*‘XGBoost + PULSCAR’*).

**Table 1:** List of diseases/conditions with the number of positive and unlabeled genes and features.

Disease	UMLS_CUI	Positive genes	Unlabeled genes	Number of features
Bipolar Disorder	C0005586, C0236780	1,479	8,660	430
Coronary Artery Disease	C0010068	1,869	8,286	505
Diabetes melitus	C0011849, C0011860, C0011854	2,428	7,734	548
Diabetic Neuropathies	C0011882	645	9,425	297
Hodgkin Disease	C0019829	1,102	9,033	391
Hyperaldosteronism	C0020428	999	9,140	365
Kidney failure	C0035078	2,276	7,886	524
Malaria	C0024530	1,280	8,860	436
Parkinson Disease	C0030567	1,964	8,189	503
Pulmonary Edema	C0034063	1,718	8,425	467
Raynaud Disease	C0034734	686	9,427	318
Schizophrenia	C0036341	2,807	7,364	580

**Table 2:** α values estimated by the PULSCAR method for each disease/condition used to test the KG2ML pipeline. Recall was computed using only labeled positive genes for both models. *‘XGBoost only’:* models were trained and tested with 5-fold cross-validation using labeled positives as class 1 and unlabeled as class 0. *‘XGBoost + PULSCAR’:* models were trained and tested with 5-fold cross-validation using labeled and PULSCAR-imputed probable positives as class 1 and probable negatives as class 0.

Disease	Estimated alpha (95% CI)	Recall (95% CI) XGBoost only	Recall (95% CI) XGBoost + PULSCAR
Bipolar Disorder	0.3368 (0.3130, 0.3606)	0.4524 (0.4502, 0.4546)	0.6122 (0.5884, 0.6360)
Coronary Artery Disease	0.3471 (0.3208, 0.3734)	0.4824 (0.4803, 0.4844)	0.6292 (0.6103, 0.6482)
Diabetes Mellitus	0.2700 (0.2552, 0.2849)	0.5005 (0.4992, 0.5019)	0.5975 (0.5839, 0.6111)
Diabetic Neuropathies	0.3462 (0.3021, 0.3903)	0.3466 (0.3430, 0.3501)	0.6371 (0.6088, 0.6653)
Hodgkin Disease	0.4356 (0.4002, 0.4711)	0.4202 (0.4174, 0.4230)	0.6840 (0.6636, 0.7044)
Hyperaldosteronism	0.4161 (0.3715, 0.4608)	0.4566 (0.4538, 0.4595)	0.6945 (0.6648, 0.7242)
Kidney Failure	0.3054 (0.2892, 0.3217)	0.4856 (0.4845, 0.4868)	0.6124 (0.5901, 0.6348)
Malaria	0.4273 (0.4148, 0.4397)	0.4635 (0.4610, 0.4661)	0.7177 (0.7076, 0.7277)
Parkinson Disease	0.4309 (0.3912, 0.4708)	0.4847 (0.4834, 0.4861)	0.7061 (0.6793, 0.7329)
Pulmonary Edema	0.3487 (0.3014, 0.3959)	0.4753 (0.4735, 0.4771)	0.6201 (0.5894, 0.6509)
Raynaud Disease	0.3263 (0.2947, 0.3578)	0.4269 (0.4241, 0.4297)	0.6613 (0.6408, 0.6817)
Schizophrenia	0.1514 (0.1235, 0.1792)	0.5171 (0.5161, 0.5181)	0.5483 (0.5325, 0.5641)

**Table 3:** Comparison of α estimated by SCAR-based methods: PULSCAR, DEDPUL, KM1, KM2, and TiCE. Estimates from the KM and TiCE methods are based on 20 iterations, while those from DEDPUL and PULSCAR are based on 40 iterations.

Disease	PULSCAR	DEDPUL	KM1	KM2	TiCE
Bipolar Disorder	0.3368 (0.3130, 0.3606)	0.5142 (0.5022, 0.5261)	0.4547	0.5255	0.4029 (0.3894, 0.4165)
Coronary Artery Disease	0.3471 (0.3208, 0.3734)	0.4760 (0.4616, 0.4904)	0.3702	0.4854	0.3950 (0.3825, 0.4074)
Diabetes Mellitus	0.2700 (0.2552, 0.2849)	0.4633 (0.4513, 0.4753)	0.3478	0.4547	0.3790 (0.3659, 0.3922)
Diabetic Neuropathies	0.3462 (0.3021, 0.3903)	0.5391 (0.5220, 0.5562)	0.5193	0.5489	0.4453 (0.4239, 0.4668)
Hodgkin Disease	0.4356 (0.4002, 0.4711)	0.5239 (0.5145, 0.5332)	0.4627	0.5375	0.4451 (0.4343, 0.4558)
Hyperaldosteronism	0.4161 (0.3715, 0.4608)	0.4844 (0.4772, 0.4916)	0.4380	0.4995	0.4443 (0.4308, 0.4580)
Kidney Failure	0.3054 (0.2892, 0.3217)	0.4391 (0.4289, 0.4493)	0.3702	0.4781	0.4039 (0.3939, 0.4139)
Bipolar Disorder	0.3368 (0.3130, 0.3606)	0.5142 (0.5022, 0.5261)	0.4547	0.5255	0.4029 (0.3894, 0.4165)
Malaria	0.4273 (0.4148, 0.4397)	0.4854 (0.4765, 0.4942)	0.4201	0.4995	0.4497 (0.4362, 0.4633)
Parkinson Disease	0.4309 (0.3912, 0.4708)	0.4389 (0.4250, 0.4529)	0.3809	0.4781	0.4105 (0.3978, 0.4231)
Pulmonary Edema	0.3487 (0.3014, 0.3959)	0.4923 (0.4823, 0.5023)	0.4202	0.4995	0.3681 (0.3579, 0.3783)
Raynaud Disease	0.3263 (0.2947, 0.3578)	0.4810 (0.4732, 0.4888)	0.4627	0.5128	0.3991 (0.3797, 0.4185)
Schizophrenia	0.1514 (0.1235, 0.1792)	0.3877 (0.3810, 0.3945)	0.2547	0.4202	0.3216 (0.3127, 0.3305)

**Table 4:** Top 15 genes with the highest calibrated probability of association for each of 12 diseases, as estimated by the PULSCAR method. Notably, several of these genes are associated with their respective diseases, even in the absence of explicit links in the knowledge graph.

Gene	Calibrated probability	Associated with bipolar disorder?
XPC	0.8870	No
ZBTB16	0.8754	Yes
SPOP	0.8734	No
STAT6	0.8730	Yes
MRE11	0.8705	Yes
GJC2	0.8697	Yes
LIMK1	0.8695	Yes
RFC2	0.8677	No
MLXIPL	0.8673	Yes
LAMC2	0.8655	Yes
PKP2	0.8647	Yes
PRKCD	0.8602	Yes
NCF1	0.8592	Yes
DDR2	0.8588	Yes
PICALM	0.8571	Yes
Gene	Calibrated probability	Associated with diabetes?
PRKN	0.8967	Yes
FASLG	0.8896	Yes
RAD54B	0.8877	No
CCND1	0.8869	Yes
KRAS	0.8798	Yes
RAF1	0.8764	No
IFNG	0.8762	Yes
LAMC2	0.8735	No
BCL10	0.8727	Yes
TERT	0.8714	Yes
SPOP	0.8660	Yes
KMT2D	0.8654	Yes
STAT4	0.8646	Yes
CC2D2A	0.8609	No
MRE11	0.8600	Yes
Gene	Calibrated probability	Associated with hodgkin lymphoma?
SLC2A3	0.8704	Yes
PKHD1	0.8700	No
FASLG	0.8699	No
VEGFC	0.8693	Yes
PEX11B	0.8664	No
LRP5	0.8610	Yes
BMI1	0.8609	No
IARS2	0.8554	No
XPA	0.8520	Yes
NTRK1	0.8458	Yes
NUP214	0.8451	Yes
RASGRP1	0.8451	Yes
PTPRJ	0.8448	No
GJC2	0.8435	No
DCX	0.8415	No
Gene	Calibrated probability	Associated with kidney failure?
ATM	0.9122	Yes
FGFR3	0.9066	Yes
KEAP1	0.9007	Yes
PIK3R1	0.8965	No
FASLG	0.8964	Yes
CCBE1	0.8909	No
IKBKG	0.8907	No
FBN1	0.8871	Yes
ADAMTS2	0.8833	No
LRRC8A	0.8829	No
AKT1	0.8802	No
RAD51C	0.8789	No
ZBTB16	0.8775	No
RB1	0.8754	No
PRKN	0.8748	No
Gene	Calibrated probability	Associated with Parkinson’s Disease?
TP63	0.9373	No
APC2	0.9333	No
DDB2	0.9315	No
SLC22A18	0.9297	No
FASLG	0.9265	Yes
F2	0.9256	No
CASP8	0.9241	Yes
TMEM237	0.9234	No
BUB1B	0.9227	Yes
NTRK1	0.9172	Yes
ERCC4	0.9159	No
SOX5	0.9159	No
FOXP3	0.9159	Yes
TRPV3	0.9153	Yes
GJC2	0.9148	Yes
Gene	Calibrated probability	Associated with raynaud’s syndrome?
SRC	0.7545	No
PLA2G2A	0.7495	Yes
GP1BB	0.7301	No
ASCL1	0.7298	No
ZNF408	0.7291	No
UFD1	0.7227	No
SEC24C	0.7203	No
GPR35	0.7197	Yes
GNPTAB	0.7168	No
OGG1	0.7114	Yes
JMJD1C	0.7101	No
TBX1	0.7087	No
ZFP57	0.7073	No
CISH	0.7060	No
CUX1	0.7050	No
Gene	Calibrated probability	Associated with coronary artery?
CCND1	0.9195	Yes
DDB2	0.9040	Yes
HABP2	0.9003	Yes
FGFR3	0.8917	Yes
SLC2A3	0.8913	Yes
MAP3K8	0.8861	Yes
RAD54B	0.8832	No
BBS1	0.8829	Yes
KMT2D	0.8779	Yes
JAK2	0.8772	Yes
FASLG	0.8768	Yes
PROC	0.8761	Yes
TBL2	0.8684	Yes
TERT	0.8680	Yes
EIF2AK3	0.8678	Yes
Gene	Calibrated probability	Associated with diabetic neuropathy?
DLC1	0.5709	Yes
PKP2	0.5413	No
LRRK2	0.5407	Yes
SDHAF2	0.5401	No
TMEM127	0.5366	No
SLC25A11	0.5356	Yes
BAP1	0.5354	Yes
KIF1B	0.5331	Yes
NR2F2	0.5298	No
KEAP1	0.5298	Yes
CUX1	0.5280	Yes
GATA4	0.5275	No
KMT2D	0.5260	No
FANCE	0.5258	No
SLC22A18	0.5255	No
Gene	Calibrated probability	Associated with hyperaldosteronism?
LAMB3	0.8770	No
DDR2	0.8677	No
HNF1A	0.8548	Yes
HSD11B2	0.8543	Yes
NFKB2	0.8531	Yes
PRKCD	0.8442	No
BMI1	0.8431	Yes
CYP21A2	0.8311	Yes
GPC4	0.8310	Yes
DOCK8	0.8308	No
GPR35	0.8270	No
SEC31A	0.8269	No
RAF1	0.8238	Yes
FGFR3	0.8234	Yes
ARL6	0.8218	No
Gene	Calibrated probability	Associated with malaria?
XPA	0.9457	Yes
NTRK1	0.9451	No
HTT	0.9440	Yes
NFKB1	0.9429	Yes
DDB2	0.9382	No
CACNA1C	0.9381	No
PLA2G2A	0.9326	Yes
TFE3	0.9310	No
BCL10	0.9304	No
RAD51C	0.9257	No
TBL2	0.9227	No
F10	0.9202	Yes
TTC37	0.9181	No
LIMK1	0.9154	No
FASLG	0.9129	No
Gene	Calibrated probability	Associated with pulmonary edema?
RAD54B	0.8654	Yes
COX7B	0.8613	Yes
LAMB3	0.8595	No
NOTCH3	0.8486	Yes
KCNT1	0.8443	No
ERCC3	0.8428	No
AXIN2	0.8374	No
FERMT1	0.8351	No
XPA	0.8338	Yes
FGFR3	0.8335	Yes
SCNN1G	0.8275	Yes
SLC18A3	0.8257	No
NR2F2	0.8218	Yes
KEAP1	0.8214	Yes
CCND1	0.8168	Yes
Gene	Calibrated probability	Associated with Schizophrenia?
PRKG1	0.6469	Yes
SLC6A19	0.6446	Yes
GJC2	0.6364	Yes
DOCK8	0.6251	Yes
SETD2	0.6226	Yes
FOXE3	0.6207	No
BCL10	0.6202	Yes
ANAPC1	0.6155	Yes
HNF1A	0.6126	Yes
TRPV3	0.6042	Yes
ERBB2	0.6039	Yes
SCNN1G	0.6037	Yes
RFC2	0.5921	Yes
PTPN12	0.5919	Yes
SOX18	0.5909	Yes
