# Supplementary material for: KG2ML: Integrating Knowledge Graphs and Positive Unlabeled Learning for Identifying Disease-Associated Genes
Source: medRxiv. 2025 Mar 17:2025.03.17.25323906. Preprint. [Version 1] doi: 10.1101/2025.03.17.25323906 (PMC11957101; doi:10.1101/2025.03.17.25323906)
Supplement: Supplement 1 [file media-1.zip › KG2ML_paper_SupportingInformation.docx]

**KG2ML: Integrating Knowledge Graphs and Positive Unlabeled Learning for Identifying Disease-Associated Genes**

Praveen Kumar^1^, Vincent T. Metzger^1^, Swastika T. Purushotham^1^, Priyansh Kedia^1^, Cristian G. Bologa^1^, Christophe G. Lambert^1^, Jeremy J. Yang^1^

^1^University of New Mexico (UNM), School of Medicine, Department of Internal Medicine, Translational Informatics Division, Albuquerque, New Mexico, USA

**Supporting Information**

**Figure 2 Cypher Queries:**

Chronic heart failure in DDKG (Panel A):

MATCH (d:Concept )-[dc: CODE]-(d1:Code {SAB:'SNOMEDCT_US'})-[pt:PT]-(e:Term)

MATCH (c:Concept )-[cc: CODE]-(c1:Code {SAB:'PUBCHEM'})

MATCH (g:Concept )-[gc: CODE]->(g1:Code{SAB:'HGNC'})

MATCH (d)-[r:indication {SAB:'IDGD'}]-(c)-[r1]-(g)

WHERE e.name="Chronic heart failure"

RETURN d as diseaseConcept, dc as diseaseCode, d1 as snomedCode, pt as diseaseTerm, e as diseaseName,

c as compoundConcent, cc as conceptCode, c1 as pubchemCode, g as geneConcept, gc as geneCode, g1 as hgncCode, r as relIndication, r1 as activityName

UNION

MATCH (d:Concept )-[dc: CODE]-(d1:Code {SAB:'SNOMEDCT_US'})-[pt:PT]-(e:Term)

MATCH (c:Concept )-[cc: CODE]-(c1:Code {SAB:'PUBCHEM'})

MATCH (p:Concept )-[pc: CODE]-(p1:Code {SAB:'UNIPROTKB'})

MATCH (d)-[r:indication {SAB:'IDGD'}]-(c)-[r1:bioactivity]-(p)

WHERE e.name="Chronic heart failure"

RETURN d as diseaseConcept, dc as diseaseCode, d1 as snomedCode, pt as diseaseTerm, e as diseaseName,

c as compoundConcent, cc as conceptCode, c1 as pubchemCode, p as geneConcept, pc as geneCode, p1 as hgncCode, r as relIndication, r1 as activityName

**Nodes**: 1,077

**Relationships**: 13,150

Chronic heart failure in Condensed-KG (Panel B):

MATCH (d:Disease )-[r:indication ]-(c:Compound )-[r2]-(a:Gene)

WHERE d.node_label = "Chronic heart failure"

RETURN d as DiseaseName, r as relIndication, c as CompoundName, r2 as activityName, a as GeneName

UNION

MATCH (d:Disease )-[r:indication ]-(c:Compound )-[r1:bioactivity]-(b:Protein)

WHERE d.node_label = "Chronic heart failure"

RETURN d as DiseaseName, r as relIndication, c as CompoundName, r1 as activityName, b as GeneName

Nodes: **214**

Relationships: **460**
